# Actualising power sharing in community-led initiatives: Insights from community-based organisation leaders in Chicago, USA

**DOI:** 10.1002/hpm.3702

**Published:** 2023-08-29

**Authors:** Alexis Grant, Jeni Hebert-Beirne, Saria Lofton, Brenikki Floyd, Emily Stiehl, Sage Kim

**Affiliations:** 1 WestEd, San Francisco, California, USA; 2 Community Health Sciences, University of Illinois at Chicago School of Public Health, Chicago, Illinois, USA; 3 University of Illinois at Chicago College of Nursing, Chicago, Illinois, USA; 4 Health Policy and Administration, University of Illinois at Chicago School of Public Health, Chicago, Illinois, USA

**Keywords:** Chicago, collaboration, community-based organisations, community-led, local health department, power, USA

## Abstract

There is an increasing call for a governmental organisations such as local health departments and federal health and human service agencies to partner with community based organisations (CBOs) for health promotion. There is a large body of literature suggesting that CBOs need capacity building or empowerment to do this work, but less literature about the necessary culture shift at governmental organisations who fund public health work. This study aimed to examine the knowledge, attitudes, and beliefs of CBO leadership who do not want to partner with state funders, and understand which structures and practices demonstrate power-sharing in a community-led approach. We conducted six interviews with community-based organisation leaders and conducted a thematic analysis and a secondary, inductive discourse analysis of the transcripts to analyse why organisations chose not to apply for a government funded initiative and how they talked about power-sharing for community-led public health. Themes about the decision for CBOs to apply to the public health funding initiative: how it related to the CBO’s scope of work, meeting the needs of the community, having the technical capacity, and cross-cutting themes of putting the community first and having a long-term positive impact. Organisations rejected the opportunity for this funding due to poor fit, even if they could fulfil the scope of work. A community-led approach was described as one that includes the government giving up control, creating spaces for meaningful participation and power-sharing, and systems demonstrating trust in CBOs. These findings reiterate that in order for public health to be community-led, there needs to be system-wide transformation and intentional investment that supports an infrastructure for community-led public health. State funders can learn from practices in trust-based philanthropy, such as flexible funding and reporting requirements. The results of this study can support the wider participation of CBOs in collaboration with state actors, maximising the transformative potential of collaboration, ultimately transforming power structures and advancing health equity.

## 1 | INTRODUCTION

There is an increasing call for a governmental organisations such as local health departments (LHDs) and federal health and human service agencies to use a community-engaged public health approach because participatory processes allocate resources more efficiently for health equity. ^1–4^ There are many models and tools that describe community engagement occurring along a continuum from community-informed to community-led, ^5–9^ with one end of continuum associated with more authoritarianism and lack of accountability, while the other is associated with more democratic and egalitarian approaches. ^10,11^ A community-led approach is one that is community-owned and where community priorities are defined and implemented by the community, who are in control of all the resources, parameters, and decisions. ^9^ Community-led approaches are marked by strong community leadership, final decision-making at the community level in which the communities may consult with external partners for technical questions, and when the outcomes reflect the needs and desires of the community. ^12,13^

Since the turn of the century, the role of the state has shifted from governing through direct forms of control to collaborative governance, which brings public and private actors together using particular processes like forums, to establish laws and rules for the provision of goods. ^14^ This shift is seen in various countries across North America, Australia, and Europe. Collaborative governance designates a specific role of state actors like LHDs and non-state actors like community-based organisations (CBOs), and all these stakeholders should work together using two-way communication and shared responsibility for outcomes. With the shift to collaborative governance, cross-sector collaboration is more common, as is a government institution contracting out a service that was previously conceptualised as their own responsibility. Collaborative governance gives community institutions a role because they are seen to have capacity for social capital and community cohesion; improve service delivery through having a greater voice in planning and monitoring; meet local needs through delivering their own services; and address concerns about the democratic deficit through re-engaging citizens with government institutions. ^15^

The shift to collaborative governance in The United States public health system can be seen in the Centers for Disease Control and Prevention’s recent 2020 revisions to 10 essential public health services (EPHS) framework. ^16^ Since its conceptualisation in 1994, the EPHS framework has created shared language and understanding about the roles and responsibilities of the public health system been used to identify national public health performance standards, develop accreditation criteria for the Public Health Accreditation Board (for LHDs) and the Council on Education for Public Health (programs and schools), and guide LHD’s structure, policies, and approaches. ^16^ The 2020 revision places equity situated at the centre of the EPHS, making collaboration with communities essential to carrying out EPHS.

Collaborative governance is commonly operationalised by state actors engaging with communities through CBOs. CBOs are broadly defined as institutions controlled within a community that contribute to community capacity in a variety of ways such as cultural organisations, communications organisations, faith-based organisations, and civic organisations that build individual assets in the community. ^17^ CBOs are good partners for community health because they can serve as proxies for community voice and they are trusted by the community, ^18–20^ both of which are important to develop and implement relevant and culturally appropriate interventions and programs.

Governmental organisations typically engage communities on the lower ends of the community engagement spectrum, collaborating with CBOs to fulfil roles in service delivery, public policy process, and governing. The typical community engagement processes for state actors is to create an initiative and issue a call for proposals and then invite community organisations to participate in the established initiative. This process is seen in various settings at local and federal levels. For example, in a systematic review of Environmental Protection Agency Request for Applications from 1997 to 2013, only 16% discussed elements of community engagement, and researchers saw that community participation was the most frequently discussed level of community engagement—which is still low on the community-engagement spectrum. ^12^ Low levels of community involvement is problematic because it gives a voice to the community but no power, ^15,21–23^ it can tokenise partners, ^10,15,21,22^ and these processes can delegitimize the community’s own self-organisation, skills, and expertise. ^21,24^

While the literature points to the need for public health system transformation to promote health equity, the path forward for state actors to share power to support community-led public health is unclear. Existing frameworks are helpful for understanding conditions for collaboration or predictors of successful collaborations, but do not necessarily explain what is important for CBO leadership when considering entering a collaboration with a state actor. ^2,6,8,14^ Little is known about how these decisions are made, highlighting a gap in understand how CBO stakeholders could be more involved in the public health system. Using a qualitative, case study approach, this study aims to examine the knowledge, attitudes, and beliefs of CBO leadership who do not want to partner with state funders, and understand which structures and practices demonstrate power-sharing in a community-led approach, from the perspective of smaller, grassroots organisations that do not readily want to participate in LHD-led initiatives.

## 2 | METHODS

This study uses a specific public health initiative responding to the COVID-19 pandemic from Chicago as a case study. Chicago, Illinois is a major city in the United States with over 2.5 million residents. The initiative called for dozens of Chicago-based non-clinical not-for profit CBOs to collaborate with the LHD to rapidly hire, train, and manage at least 16 individuals that would make up Chicago’s contact tracing corps, as a way to invest in communities disproportionately impacted by COVID-19 while creating a public health workforce representative of the residents. The City allocated 24.6 million dollars directly to CBOs (up to $896,100 per organisation). The initiative began in September 2020 and was ultimately extended until 2022. The case study approach conducts a detailed examination of a single case of a phenomenon. This approach can be particularly useful for investigating nonlinear, complex, and context-specific processes like collaboration. ^25^ Using a specific initiative as a case study for this research allows for an in-depth exploration of the local and historical context surrounding the initiative and yields hypotheses that can be further explored with a larger number of cases.

We explored how CBO leaders conceptualise community-led public health by conducting a series of virtual 90-min semi-structured key informant interviews with leadership from CBOs that did not respond to the initiative’s request for proposals (RFP). The goal of the key informant interviews was to understand knowledge, attitudes, and beliefs of CBO leadership along three domains-the context of the RFP opportunity, congruence between the goals and values of their organisations, and their own organisations’ capacity and readiness. This study is a multi-method analysis of interview data to understand CBO leaderships’ decisions not to apply for governmental funding and their conceptualisation of power sharing in a community-led initiative.

### 2.1 | Community engaged research mechanism

The study was informed by a 3-member advisory board that was convened at the start of the study to address potential biases such as assumptions and preconceived ideas that come with people being studied but not those doing the studying. ^26^ Advisory board members were intentionally recruited to bring diverse experiences as non-profit executives, board members, and technical assistance providers who each hold at last a master’s in public health degree. The advisory board met three times and provided feedback on the recruitment flyer, interview protocol, and interpretation of findings. Members of this advisory board were compensated for their time, at $50 per hour.

### 2.2 | Sampling and recruitment

A recruitment flyer was distributed on listservs with funders, research networks, and through word-of-mouth referrals from the author and the advisory board members. Interested individuals were assessed for eligibility in the study via a brief survey distributed over email. Eligible participants were over 18 years old, employed at a not-for-profit, non-clinical CBO in the city that did not apply for the initiative; and had a position where they participated in decision-making related to responding to RFPs. After assessing eligibility and reviewing study information, interested individuals were enroled for participation in the study and scheduled for an interview according to their availability. The number of interviews was determined based on saturation. ^27^ Initially, six interviews were scheduled based on the order that interviewees enroled in the study. After the fourth interview, we determined that no further interviews were necessary to schedule because there was not a wide variation in interviewees’ responses.

### 2.3 | Pre-interview activity

Each participants’ interview was informed by a pre-interview elicitation exercise. Elicitation exercises can be considered part of a visual method and can be useful to establish meaning from participants’ own created documents. ^28,29^ Participants were instructed to read a prepared excerpt of the RFP and annotate words or phrases that stand out to them as important, informs their thoughts on whether or not to apply, or raises questions or concerns. The annotations were reviewed prior to the interview. Participants who did not complete the annotation prior to the interview were still elicited by the interviewer reading sections of the RFP word-for-word or screensharing, whichever was preferred by the participant.

### 2.4 | Key informant interviews

A semi-structured interview guide was structured to cover the domains that influence a CBO’s decision to apply to funding, based on a review of the literature: the context, the congruence, the organisation’s capacity and readiness. The interview guide was designed to elicit insight on the participants’ knowledge, attitudes, and beliefs about their organisation’s function and role in the public health system, both historically and currently. Specifically, participants described their process of reading the initiative’s RFP and what aspects discouraged them from applying.

The interviews were conducted on zoom and audio and video recorded with automatic transcription. At the end of the interview, participants were asked to complete a brief demographic questionnaire that captured their gender, race/ethnicity, and highest level of education completed. In exchange for their time, participants were compensated with a $100 visa gift card, electronically delivered to their preferred email at the completion of the interview. Interviews were conducted in March and April 2022. The procedures of this study were reviewed by the advisory committee and also approved by the University of Illinois Chicago Institutional Review Board.

### 2.5 | Field notes

At the time of the study, the first author was research assistant working with CBOs that received the funding. As a participant-observer with prolonged engagement with the subject matter, ^30^ she maintained field notes since August 2020. ^31^ The field notes included observational notes about what happened during meetings with project partners and funded CBOs and what people communicated as well as theoretical notes, capturing themes and ideas raised during the field experience. ^32^ The notes were organised chronologically in a single notebook and were later typed up and imported to the qualitative analysis software for reference.

### 2.6 | Analysis

A critical, social constructivist lens guided this research, reflecting how each individuals’ realities are informed by their own context and experiences. ^33^ Data was analysed using Dedoose (2002), and participants’ annotations were incorporated into Dedoose as memos and later linked to themes as they were constructed and revised, as a method of triangulation. ^30^

To understand participants attitudes, knowledge, and beliefs, we used a reflexive thematic analysis approach, in which themes are conceptualised as meaning-based patterns and as the output of coding. ^34^ To understand power-sharing in community-led initiatives, a secondary inductive discourse analysis was conducted with attention to how CBO leaders talked about power-sharing occurs in public health collaborations. ^35,36^ Discourse analysis is an analytic approach that is useful for engaging with data using a critical view, uncovering meaning from what people say and how they call for action. ^36^ This method builds on the idea that speech is a form of action-the way individuals talk about something has consequences from which we can make inferences about power relations. ^35^ To conduct a discourse analysis, the data was approached with the following questions in mind ^35^:

- What discursive resources are used to describe what community-led looks like?
- What assumptions underpin what is said about public health system and actors?
- What kind of discursive resources are being used to construct meaning?
- What are potential consequences or implications of the discourses that are used?

After identifying main insights, field notes were triangulated to compare with insights and expound descriptions. After conducting each analysis, member checking was conducted with advisory board members. Member checking is taking ideas back to participants for their confirmation. This process supports the credibility and confirmability of findings by providing insights from CBO leaders that guide the study’s analysis and interpretation. This process can also serve to elaborate categories and enquire to what extent they fit participants’ experience. ^30^

## 3 | RESULTS

This study examined existing qualitative data from six key interviews conducted with executive directors and programme directors working in Chicago CBOs. Table 1 shows interviewee characteristics. Although some participants were relatively new to their role (i.e. ID 1), they had decades of experience at other CBOs as both programme staff and in leadership roles. Interviewees belonged to organisations that had programs in areas of civic engagement, advocacy, violence prevention, youth development, case management, and community resource centres.

Across the interview data, RFP annotations, and fieldnotes, three primary themes emerged that inform why CBOs did not apply to the city-funded public health initiative: it was outside of organisation’s scope of work, it was not responsive to community needs, and technical and administrative concerns with the funding opportunity. In addition, two cross-cutting themes emerged: that the community comes first and that there were doubts about long-term impact. In talking about their decision to not apply to the funding RFP, interviewees described three inter-connected practices of power-sharing in a community-led approach triangulation of interview data and reflective fieldnotes: giving up control, created spaces, and demonstrating trust in CBOs.

### 3.1 | Outside of our scope of work

First, participants talked about how the RFP did not describe the work that they do. Interviewees said they are looking for grants that ‘drive [their] work’. The RFP describes the initiative as one where CBOs ‘serve as local employers’ and said the initiative was about ‘investing in communities most impacted by health inequity’. But as participants read the RFP, they took away that the opportunity was a healthcare workforce development initiative and questioned why healthcare organisations were ineligible to apply for the opportunity. Interviewees said ‘that’s not my area of expertise’ and ‘we’re not in the business of contact tracing’. With healthcare being outside of CBOs’ scope of work, there was concern that contact tracers would not be sufficiently professionalised for the job, since organisations did not have content knowledge to train a healthcare workforce:

My understanding is contact tracers just have very specialized training, and often, bachelors if not graduate level degrees in public health, to train them for this type of work. […] We were not looking to build up a contact tracing workforce.

Interviewees spoke of seeing the value of contract tracing work but called it ‘a stretch’ as it related to their organisations’ mission and goals. Furthermore, participants recognised that creating a contact tracing workforce was a big ask of their organisations and saw that ‘the funding really doesn’t suffice in terms of the demand, the hands-on that’s gonna be required to roll it out’.

### 3.2 | Not responsive to community needs

A second major theme was that the initiative as described in the RFP did not address tangible needs of CBOs’ client base. At the start of the pandemic, CBOs saw people losing their jobs, losing family members, and struggling to pay their bills. A major concern, then, was how people could get immediate relief. One interviewee stated that the initiative ‘doesn’t […] fit within our typical model of being very client-led and responsive to client needs’ In order for the initiative to be congruent with CBO’s dedication to being responsive to the community needs, interviewees suggested that the funding opportunity should include money to give to individuals or put towards relief resources:

You’re supposed to then point [people] to the website or give them a brochure or give them a list of phone numbers that they should call and to take? That’s not the way [our organization] operates. We were on the ground trying to deliver and being engaged with people around the material needs. [It wasn’t feasible] adding the responsibility of being a contact tracer to that, without any additional resources or material supports to get people the resource that they need to stay at home.

More broadly, participants noted if the funder puts a boundary around the groups or areas that can be served by the funding, it could potentially limit the work the CBO could do. Providing insight into their CBOs’ experience with grants that do not meet community needs, participants spoke about how many grants are prescriptive about what services to provide and to whom:

[The funder is] gonna give us [money] through [a funnel] and [tell us,] “this is how you spend it.” [It’s] like, chicken wings and fish don’t solve everything for us. There’s more stuff[…] Sometimes the teams are like saying, “can we do this?,” and I’d be like, “Not on this grant. We’ll find it somewhere else.”

#### 3.2.1 | Technical and administrative concerns

Another theme was that there were technical and administrative concerns related to unanswered questions about the RFP. Participants were frustrated by the lack of detail in the scope of work, including how expectations would change over time and how contact tracers would be trained. One interviewee expressed concern around what new systems they would need to adopt and ‘how easy it is. What would it be for us to train our workers to work within that system?’ There was also concern around unstated outcome metrics, and without these details, it was difficult for CBOs to evaluate if they could fulfil the scope of work.

In addition, there were concerns about organisational capacity to apply for the grant and begin activities in a short period of time. The application window was less than a month, which participants thought was a quick turnaround and a challenge since there was only one advertised technical assistance session. Participants also voiced concern with the capacity to hire 16 people in less than a month when the contract started, and with the grant operating on a reimbursement structure. For three CBOs, this meant dramatically expanding staff size and it would be a challenge for these smaller organisations to pay wages using existing funds while they waited to be reimbursed. One interviewee suggested that ‘if the grant can be paid at the assigning of the agreement, or partially, that would be better than the reimbursement structure’. This was also reflected in participant annotations, as one interviewee wrote ‘concern: do we have the cash on hand to float the build up of this level of staffing in a new programme?’

### 3.3 | Cross-cutting insight: The community comes first

Interviewees repeatedly described their work as being grounded in the needs of the community. All of the organisations were founded with a core mission to address a specific, identified need in the community, and the trajectory of each organisation is based on finding resources to meet those identified needs. Participants reflected that if they were to expand their organisational scope for the scope of work described in the RFP, it would impact their capacity to do other essential work:

We absolutely want to connect residents with people who are doing job training, but […] putting a bunch of capacity towards that would have taken away from our ability to continue with our core mission.

Regarding engaging in public health, participants read the RFP through the lens of how it met the needs of the community, and by extension, fulfiled their CBOs’ central mission. CBOs did not reject the notion of having a role in public health, but instead their responses drew attention to the question that perhaps public health leaders do not understand what the community needs. Although the participants represented CBOs that did not apply to the funding opportunity, these CBOs were resourceful in finding funds to meet the needs of the community during the pandemic. Participants spoke about their organisations’ response to the pandemic, such as how they created and administered needs assessment surveys and outreach phone calls to identify the needs of current and previous clients. These organisations did not acquire specific funding to perform these services but did so with their own reserve funding. Another participant organised resources among various organisations in their community, started a 24-h COVID response phone line, and began a small business assistance programme. Several organisations distributed funds, food, and personal protective equipment to residents using mutual aid strategies:

It was pretty obvious that we needed to figure out a way to start getting money funds to families. So we started our own COVID relief fund and started getting people like through a kind of a mutual fund strategy, trying to get people to donate to that fund and then figure it out that way.

As a whole, interviewees emphasised that the needs of the community came first, and only then did they consider the means to the end. Field note data raised the question if organisations would accept funds that did not align with their goals out of need, but that idea was not represented in the interviews. One participant shared, ‘if it’s completely outside of our scope of work, then it doesn’t matter how much money they’re offering’. If interviewees perceived the initiative as something that would make meaningful impact, the technical aspects were not communicated as barriers to engaging. Rather, the technical aspects are barriers that come into play when organisations are hesitant to engage, and then a long application or a lot of reporting requirements are seen as barriers to engagement.

### 3.4 | Cross-cutting insight: Doubts about long-term impact

Participants also doubted the potential impact of the initiative, with the short performance period of just a year and unclear metrics. Responding to the RFP, a participant annotated the phrase from the RFP ‘invest in communities most impacted by health inequity’ and asked, ‘how is impact defined?’

One of the goals of the initiative was to address inequities in access to healthcare, information, and health outcomes, but participants saw the initiative as ‘lots of work to scale up quickly, only to then have to let people go’. Participants stated that CBOs are open to starting new initiatives that meet community needs, but they should be sustained over multiple years. Otherwise they will be wasting resources to get a new initiative started:

You’re not going to expand for one year service for anything because you don’t want to lay people off. […] The 6 months it’ll take you to get things off the ground to only produce 6 months of work.

The short timeline is a deterrent to applying because CBOs want to apply for grants that expand their existing capacity, rather than starting a new programme area, even if it can fulfil community needs. A particular concern of this grant was that it included hiring 16 people at one time, and CBOs were concerned about what would happen to these individuals at the end of the grant:

It was just sort of like, “your funding is gonna end, and like you have trained all of these people and supported them” and like there wasn’t a clear next step […]. So I have a lot of concerns about like, even if we did have the capacity to hire and train all of those people, what’s the next step?

Especially without a guarantee of renewal, interviewees did not see this opportunity as helpful to the long-term capacity of the organisation or to the benefit of the community. As one participant stated, ‘you can’t expect the world to change on a shoestring budget. So I think that there’s a misalignment a lot of times between the amount of funding and the level of expectation of the results’. The long-term impact of the initiative was unclear, particularly as it pertained to addressing inequity in health outcomes.

### 3.5 | Power sharing: Giving up control

Interviewees thought of their organisations’ ability to make lasting change as dependent on governmental decision-makers’ ability to share power by giving up control and allowing communities to make big decisions like in policy and funding allocation. A field note entry documents an observation that CBOs are asked to focus on downstream determinants of health such providing access to vaccines, while government priorities are focused upstream activities like on disinvestment in community health centres. CBOs are not typically given the opportunity to impact the upstream factors because they are not given power to influence decision-making.

Participants linked lack of power sharing with the government maintaining control, using words like ‘guardrails’ or ‘safety’ when describing what was allowed or not allowed in collaborations. For example, one interviewee recollected that ‘whenever you would voice a concern that was outside of that parameter that was established of what’s safe to talk about there, you would quickly not be invited, or be […] ignored.’ As a result, lack of disagreement stifles the potential of what the organisations could do as innovators, as another participant reflected:

There’s no space for innovation or for something new. It’s kind of like, “this is the plan and this is what we’re gonna talk about.” Like “we’re gonna talk about vaccine events.” Not a different way of distributing vaccines.

Interviewees described how the decision-makers downtown made decisions on what should be done: ‘I always point towards the east, like in the downtown area, and then they tell us what was best for down here [on the south side], and that’s not always so.’ Another participant reflected, ‘what would it look like to really invest in public health? But you know the fact that those conversations are not allowed to happen, there lies the real point.’

Examining the discourse of rule-setting, allowable versus non allowable showed an uneven power dynamic between CBOs and the LHD decision-makers. Interviewees assume that LHDs do not want to share power because they do not create a structure for it, and the consequence is a hesitancy to participate in public health initiatives. For participants, power-sharing requires governments to share power to set agendas, start discussions, and determine boundaries. Agenda setting control and allow CBOs to co-create the agenda or set priorities. One interviewee specifically talked about the ability to ‘define a win’ for his community, while another participant gave an example of how Asian American communities were excluded from some funding opportunities, and ‘it’s unfortunate that sometimes Asian American communities are seen as not as needy. When it’s that the needs are different-- not that there are fewer needs.’

### 3.6 | Created spaces

All interviewees mentioned the figurative ‘table’ where stakeholders come together to make and implement decisions. The table was recognised as an important site because

All of [the funding opportunities are] a result of the dialogues that you know have taken place, and then it’s shaped into these opportunities for us to then step up and roll out the services and support. […The funders are] doing the best they can with the information that they can see.

The construct of a table is reminiscent of what Gaventa (2005) calls spaces of participation, which are social products that are a constructed means of control, and they provide opportunities, moments, and channels where people can be heard and act to potentially affect policies, discourses, and decisions. ^37^ Participants talked about the table idealistically, indicating that currently, the existing spaces are not as open as they should be. Participants used spatial terms such as ‘closer’ and ‘centre’ to describe the role of organisations and decision markers as working together collaboratively. One interviewee stated: ‘the people closer that are gonna be managing the people, they need to be at the table to share input.’ Another interviewee described the need to be

… serious public health people at the kind of center of it, helping to organize the space and […] create the table, for lack of a better word. I think it should be, you know, much more open than what it is. […] There’s no reason for it to ever have been exclusive. Which is what it is right now. It’s like you have to be invited to the table.

By ‘serious public health people’, this interviewee meant people with decision-making power at the city government level should be present, rather than representatives who have to get additional approval to make decisions. ‘Non-serious’ public health people at the table inevitably leads to CBOs being discouraged in the process and feeling tokenised for their participation.

### 3.7 | Demonstrating trust in CBOs

Interviewees pointed out that the government verbalises value for their organisations’ work but noted that the structure of collaborations demonstrates a lack of trust in CBOs’ work. The discourse used by interviewees showed that they were frustrated by being seen as untrustworthy partners, as they discussed proving themselves, scrutiny from funders, and restrictions or burdens put on CBOs. For example, one interviewee talked about their strengths but also how they had to ‘prove’ themselves to funders:

I think that [our model] has a potential to be a really powerful tool. And so I think to the extent that government entities can be open to innovation in a way that we don’t have to try to prove that we work within a really short window of time …

In addition to funding that could be used for administrative costs, participants saw that the administrative requirements of grants and contracts is typically overbearing and not consistent with the purpose for monitoring:

I don’t think the level of scrutiny matches the level of fraud that exists within organisations. So I feel like there could be a happy medium between “here’s money don’t tell us how to use it,” and like, “tell us how every single penny was spent.”

The use of words such as ‘scrutiny’ and ‘fraud’ reflects an unequal relationship between funders and CBOs; that CBOs need to be watched closely to ensure they do not take misuse the money. This is ironic, considering that many CBOs are established for the well-being of the community, similar to what funders’ goals are.

A result of this unwarranted surveillance is that funders take away from the work itself, as one participant shared: ‘it’s like the more restrictions you put in place, […] the more administrative burden you’re putting on them, [it] prevents them from doing their work.’

## 4 | DISCUSSION

The goal of this study was to examine the knowledge, attitudes, and beliefs of CBO leadership who do not want to partner with state funders and understand which structures and practices demonstrate power-sharing. The insights from interviewees suggest that initiatives like the one used in this case study should not assume that the government’s call will resonate with a variety of CBOs, and that they may not prioritise if and how their CBO’s collaboration will increase the capacity of the public health system. For instance, this RFP specifically called for organisations that did not do contact tracing and for organisations that were non-clinical. The funders assumed that the reasoning for this approach would be easily understood by organisations and that they would share vision of building local public health infrastructure, but that was not the case, as prominent themes included concerns about the initiative falling outside of the organisations’ scope of work and failing to address the needs of the community. Interviewees also engaged in discourse that described an unequal power dynamic between CBOs and the LHD funders. There are many tensions in this power struggle-organisations want funders to give up control so that their communities’ needs are covered, but there should also be oversight to ensure no communities are overlooked across the broader geography. This work also raises the question of if it is enough to have everyone invited to the planning table and the acceptability of only awarding contracts to the small number of organisations to carry out the work. The results of this study do not suggest a one-size-fits-all approach, but an overall strategic direction that funders should follow in all their opportunities: to create the space for CBOs to be heard, without unnecessary boundaries that ultimately affect the quality of the work. Collaboration has the potential to distribute power and responsibility to the community, but for this to happen there needs to be a shift in the traditional structures that were created to establish and separate governmental processes from citizens. ^21,22,38^

Interviewees were looking for funding that can be used to meet community needs, above all, and that opportunities should result in long-term impact instead of ‘feel better funding.’ There must be a complete culture change and socioeconomic reform that enables community-led public health. ^10,15,21,22,39^ The results of this case study show that there has not yet been a culture shift and LHDs need to share power with communities. If power from the state is delegated to CBOs in communities, community engagement could strengthen community empowerment, ^24,40^ the ownership of community interventions, ^41^ and lead to policy, systems, and environmental changes in the institutions that perpetuate health inequities. ^15^ Unfortunately, as seen in the key informant interviews, the actual practice of co-governance does not reflect the promise of being decentralised, participatory, and transformative. ^24^ These insights are not new—the need to transform how governments do public health has been raised by scholars for decades. ^39,41–43^ This study contributes to the literature by emphasising that power-sharing is non-negotiable when it comes to public health collaboration.

Structural factors that demonstrate trust is important for collaborations because it shows that organisations are respected as experts in their field. ^7,44,45^ Policies requiring how and when to use funds and the level of oversight conflict with the supposed freedom that should come with community-led efforts. Interviewee’s suggestions for flexible funding and reporting requirements coincide with examples from trust-based collaboration and trust-based philanthropy. ^7,45–47^ A trust-based approach addresses concerns around equity in grantmaking by going beyond traditional programmatic restrictions in grantmaking and places trust in organisations to use resources in ways that meet the needs of their staff, programs, and communities they serve. A trust-based collaboration might have a detailed service purchase agreement that is a living document open to modification, opportunities with more flexible funding formulae, and low level of monitoring and paperwork. ^44,45,47^ The charge here is for public health institutions to create or revise structures to be less hierarchical and less bureaucratic. This is the essence of created spaces—they are where empowerment takes place and less powerful actors can define the space and shape a healthy culture of participation. ^37^

### 4.1 | Limitations and conclusions

One limitation of this study is that it only includes 501c(3) organisations that had paid staff. Although findings of this study may be generalisable, the perspective of volunteer organisations or non-registered CBOs are not represented in the data. It is important to understand what community led public health can look like for various types of organisations such as block or neighbourhood groups, tenant associations, religious groups, volunteer groups, youth groups, and merchant associations. ^48^ Although these types of organisations are sites of innovation, much of the time, they are left out of research. ^49^ Another potential limitation of this study is that the participants brought a specific perspective as executive directors and programme directors who volunteered for an interview about community-led public health. These findings may not represent the perspectives of CBO leaders that are completely disinterested in formally engaging in public health work, or of programme staff that are in the same spaces as their leadership. Despite these limitations, these findings shed light on the ways in power-sharing can be actualised for LHDs. There must intentional efforts for governments to allow CBOs to set agendas and priorities, participate in decision making in created spaces marked by power-sharing, equity, and innovation, and treat CBOs as trusted partners that are not heavily surveilled.

## Figures and Tables

**TABLE 1:** Research participant characteristics.

ID	Role	Time in role	Gender	Race/ethnicity	Highest degree earned	Organisation programme areas	Staff size

1	Programme director	2 months	Male	Latine	High school	Violence prevention and outreach	30
2	Executive director	3 years	Female	Chinese American	Masters	Civic engagement, advocacy, outreach	7
3	Executive director and founder	9 years	Female	African American	Doctoral	Youth development, community resource centre, violence prevention	3
4	Programme director	2 years	Male	Mexican	Doctoral	Social services and community organising	50
5	Executive director	15 years	Male	White	Bachelors	Social services and community organising	50
6	Executive director	5.5 years	Female	White	Masters	Community resource centre	6

*Note:* ID 4 and 5 belong to the same organisation. Demographic variables are self-reported. Organisation programme areas and staff size were extracted from interviews.

## Data Availability

Research data are not shared.

## Highlights

- For broader participation in public health, funders should structure opportunities to community needs, clearly communicate their goals for sustainability, and build trust with community-based organisations (CBOs).
- CBOs cannot have shared power while there are exclusive spaces and guardrails set up by funders.
- In order to execute a community-led approach, funders and decision-makers must give up control by creating spaces for meaningful participation and establishing systems that they demonstrate trust.
